# A facile approach based on test strip for on-site determination of glucose in tobacco matrices

**DOI:** 10.3389/fchem.2026.1948329

**Published:** 2026-08-31

**Authors:** Kai Cai, Jinsha Ba, Jie Zhang, Ruijuan Zhao, Yunfei Ma, Yechun Lin, Changyun Zhang, Decheng Li, Weichang Gao, Kesu Wei

**Affiliations:** 1 Guizhou Academy of Tobacco Science, Guiyang, China; 2 Guizhou Provincial Tobacco Quality Supervision & Test Station, Guiyang, China; 3 State Key Laboratory of Soil and Sustainable Agriculture, Institute of Soil Science, Chinese Academy of Sciences, Nanjing, China

**Keywords:** AGREEprep assessment, glucose, matrix effect, on-site, test strip

## Abstract

**Introduction:**

Glucose in tobacco leaves acts as a vital chemical precursor that thermally degrades and reacts with amino acids during combustion and curing to enrich aldehydes, ketones, and nitrogen‐containing heterocyclic aroma compounds, thereby shaping flavor and masking harshness. However, current analytical methods for glucose quantification are limitations of high complexity, poor portability, and low eco-friendliness.

**Methods:**

A facile approach for on-site method for glucose determination in both flue‐cured and fresh tobacco leaves, based on aqueous extraction followed by test-strip reflectometric analysis, was developed.

**Results:**

Design of experiments with central composite design was employed to achieve the ideal extraction conditions, with a shaking count of 70 times and a static immersion time of 36 min. The matrix effect in the aqueous tobacco extract showed a suppression effect with relative quantitative errors below 5.0%, and the test strip’s enzymatic reaction exhibited high specificity for glucose. The developed method showed a good linear relationship over the range of 2.00–90.0 mg/L (y = 0.9952× + 0.0253, *R* ^2^ = 0.9991). The average recoveries ranged from 94.1% to 96.0% for flue‐cured tobacco and 95.0% to 97.5% for fresh tobacco leaves, with repeatability RSDs ≤ 3.86% and ≤ 4.51%, reproducibility RSDs ≤ 5.00% and ≤ 5.98%, respectively. Compared with the traditional derivatization GC‐MS method, the linear regression slope between the two methods was 0.9781 (*r* = 0.9997 and p < 0.01), and the overall AGREEprep score of test strip with reflectometry method was 0.78, further confirming both the excellent accuracy and greenness. Finally, this method was successfully applied to evaluate glucose variation among different cultivars and growth stages, demonstrating its practical applicability.

**Discussion:**

This study developed a simple, portable, and green method for the determination of glucose content in different tobacco matrices, which could provide scientific reference data for tobacco quality evaluation and physiological growth monitoring.

## Graphical Abstract

**Figure:**
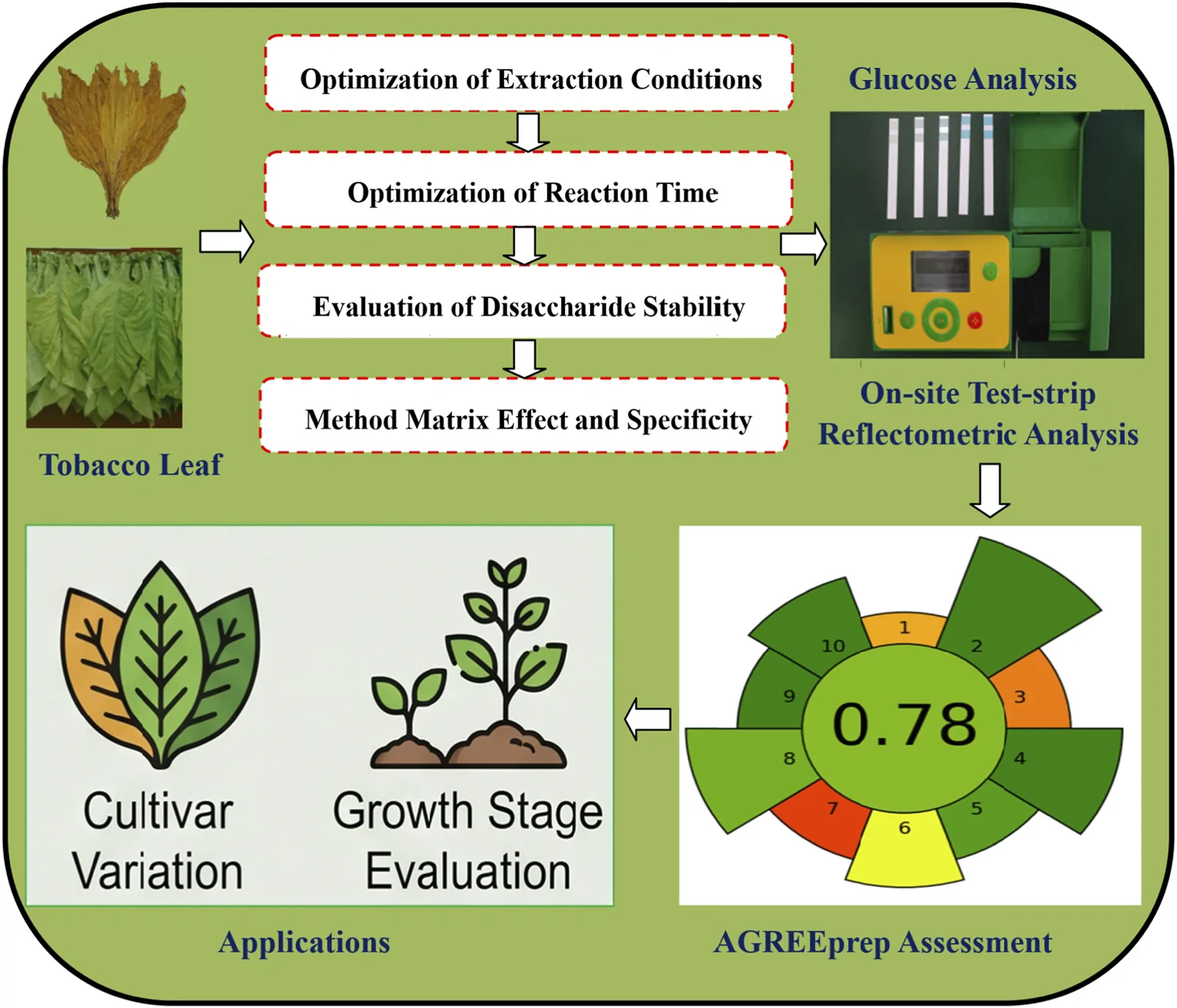

## 1 Introduction

Tobacco is rich in water-soluble sugars, including monosaccharides, disaccharides, and other soluble oligosaccharides, typically glucose, fructose, sucrose, maltose, fructo- and malto-oligosaccharides (Nagai et al., 2012). Under high thermal treatment, water-soluble sugars can undergo thermal cracking to produce new compounds like furans, furfural, ketones, and aldehydes, which serve as aroma precursors, thereby affecting the aroma and taste (Banožić et al., 2020). In addition, water-soluble sugars also reflect the tobacco’s physical characteristics. Tobacco leaves with higher sugar content tend to be softer, more elastic, and brighter in color. Glucose is a vital water-soluble sugar in tobacco leaves, generally accounting for 5%–15% of the leaf dry weight, and is one of the important factors affecting the quality of tobacco products. In cigarettes, glucose significantly enriches smoke aroma, improves the smoothness of the smoke, and imparts a distinctive flavor to tobacco products (Wang et al., 2021). It has been reported that glucose enhances the diversity of volatile components in cigarette smoke, particularly aldehydes, ketones, and *N*-heterocyclic aroma compounds, while fructose shows a comparatively weaker influence on smoke volatiles (Chen et al., 2025). Glucose also undergoes the Maillard reaction with amino acids during curing processes, generating the abundant Amadori compounds that serve as key aroma precursors (Liu et al., 2017). Within a certain concentration range, higher glucose content in tobacco leaves is associated with better leaf quality; however, excessive glucose can result in a flat taste, reduced strength and impact, poorer combustibility, and increased tar yield. Therefore, rapid and accurate on-site analysis of glucose content in tobacco is of great significance for quality evaluation.

Extraction and detection are critical steps for the quantification of glucose in tobacco leaves. Design of experiments (DoE)-based response surface methodology (RSM) is frequently used in extraction method optimization, as many parameters can affect extraction performance (Fang et al., 2026). The main advantages of RSM are its ability to save both time and resources by allowing systematic experimentation. Currently, the commonly used detection methods for analysis of glucose include UV-vis spectrophotometry, gas chromatography (GC), high-performance liquid chromatography (HPLC), and ion chromatography (IC) (Cai et al., 2012; Cai et al., 2015; Huang et al., 2018; Li et al., 2024; Li et al., 2025; Shifflett et al., 2012; Tang et al., 2007). UV-vis spectrophotometry requires the hydrolysis of glucose to furfural under acidic conditions, followed by colorimetric detection using phloroglucinol. However, various reducing sugars in tobacco can also form furfural derivatives, resulting in potential interference (Li et al., 2024). GC offers high sensitivity and rapid analysis, but glucose analysis requires tedious derivatization prior to sample injection, which complicates the procedure and limits its practical application (Becker et al., 2013). HPLC with refractive index detection provides a rapid and direct method for sugar analysis, yet it is sensitive to temperature fluctuations and suffers from relatively low sensitivity. In contrast, HPLC with evaporative light-scattering detection exhibits good stability and higher sensitivity than refractive index detection, making it suitable for the analysis of low-content sugars, but it requires time-consuming C18 or NH_2_ solid-phase extraction for sample clean-up to remove impurities and protect the chromatography column (Pang et al., 2006). IC offers some advantages such as high sensitivity, no derivatization requirement, simple operation, and avoidance of toxic reagents; nevertheless, it is characterized by longer analysis time and relatively lower analytical throughput (Peng et al., 2023). Prolonged storage of tobacco extract solutions can lead to the degradation of disaccharides, which consequently results in inaccurate quantification of glucose (Huang and Huang, 2012). Therefore, it is necessary to develop a simple and accurate method for the determination of glucose in different tobacco matrices, which could provide scientific reference data for tobacco quality monitoring.

The test strip with reflectoquant analysis system has been widely applied for on-site analysis in various matrices, including plants, food and beverages, soil, and water, and can be used to determine the content of nitrate, phosphate, potassium, calcium, ascorbic acid, hydroxymethylfurfural, and other analytes (Cai et al., 2023; Hošťálková et al., 2013; Major and Barraclough, 2003; Schmidhalter, 2005). This system offers several advantages, including easy-to-use, cost-effective, portable instrumentation, and suitability for field applications. The glucose test strip operates on the principle that glucose is oxidized to glucono-δ-lactone catalyzed by glucose oxidase; then the hydrogen peroxide generated in this reaction reacts with an organic redox indicator in the presence of peroxidase, forming a blue-green substance. The color intensity is determined by a reflectometer to assess the glucose content (Helgerud et al., 2016). Due to the enzymatic reaction, this method provides high sensitivity and selectivity. Moreover, the colorimetric reaction of the test strip does not use toxic chemical reagents, making it ecologically and environmentally friendly (Cai et al., 2023). Currently, this method has primarily been applied to the quantitative analysis of glucose in simple matrices such as fruit juices, honey, and potato (Balık et al., 2023; Helgerud et al., 2016; Xolalpa-Aroche et al., 2024); its application to complex matrices such as tobacco has not yet been reported. This method would be beneficial for on-site evaluation of tobacco leaf quality.

In this study, a glucose test strip combined with a reflectometer was employed to develop a simple, portable, accurate, and cost-effective method for the determination of glucose content in fresh and flue-cured tobacco matrices. The main purposes include: (1) optimization of the extraction conditions of on-site detection and the reaction conditions of the test strip; (2) evaluation of the matrix effects and specific selectivity under complex tobacco matrix; (3) comparison of the accuracy and greenness performance between test strip with reflectometry and derivatization GC-MS method; (4) Monitoring and evaluation of the glucose variations in different tobacco cultivars and at key growth stages. By developing a simple, portable, and green method for glucose determination in tobacco, this study aims to provide both technical and theoretical foundations for the on-site quality evaluation and physiological growth monitoring of tobacco leaves.

## 2 Materials and methods

### 2.1 Chemicals and reagents

All chemicals were of analytical grade unless otherwise indicated. The standard compounds such as glucose, sucrose, fructose, maltose, inositol, malic acid, and nicotine (purity, > 99.5%) and internal standard phenyl-*β*-D-glucopyranoside hydrate (≥ 99%) were obtained from Aladdin. The derivatization reagents of methoxyamine hydrochloride (MEOX, ≥ 98%), *N, O*-bis (trimethylsilyl) trifluoroacetamide (BSTFA, ≥ 98%), and anhydrous pyridine (≥ 99.5%) were all purchased from Merck Sigma-Aldrich. Deionized water was purified by a Milli-Q water purification system.

### 2.2 Preparation of standard solutions and samples

A total of 501.3 mg of glucose was weighed, and standard stock solutions were prepared at 50.13 mg/mL with deionized water. Working standard solutions with 2.00, 5.00, 10.0, 20.0, 50.0, and 90.0 mg/L were prepared freshly before use. Internal standard solutions of phenyl-*β*-D-glucopyranoside were prepared at 10.25 mg/mL with acetonitrile-water (*v/v* 1:1). Sucrose and maltose stock solutions were prepared at 25.12 and 2.08 mg/mL, diluted appropriately, and used for the GC-MS quantitative evaluation of the hydrolysis degree.

To compare the method accuracy, twenty flue-cured tobacco samples representing a broad range of glucose content (about 4.0%–12.0%) were selected. In addition, four cultivars (Nanjiang 3, Yunyan87, K326, and Bina 1) were grown in field plots at a tobacco planting base in Fuquan city, Guizhou province, China. The field design was a completely randomized block design with each cultivar. Seedlings were grown in a greenhouse, transplanted to the field on 28 April 2024, and field management was conducted uniformly according to the local standard practices for high-quality tobacco production. The fresh tobacco samples (middle leaves: the 9–10th leaves from the bottom) were collected with the half-leaf method at seven growth stages: rosette, vigorous growth, budding, 10 days after budding, 20 days after budding, 30 days after budding, and maturing. One part was directly analyzed on-site by test strip with reflectometry method; the remaining part was immediately placed in liquid nitrogen, transported to the laboratory, and dried in a vacuum freeze dryer (SIM Inc., DE, United States) to obtain freeze-dried fresh tobacco, which was used for derivatization GC-MS method. The mature leaves were later harvested and cured in a barn with circulating heated air. The flue-cured tobacco samples (C3F) were collected from four cultivars and then oven-dried at 60 °C for 48 h and finely ground to pass through a 40–60 mesh sieve prior to analysis.

### 2.3 Extraction procedures

Fresh tobacco leaves: A 1.00 g portion was sampled from the middle section of the leaf blade and accurately weighed into a mortar. 2 mL of deionized water was added for extraction, and the mixture was ground to a homogenate. The homogenate was quantitatively transferred with water into a 100 mL volumetric flask and made up to volume. A 3.0 mL aliquot of the resulting extract was filtered through a hydrophilic membrane filter into a 2 mL centrifuge tube to obtain the sample solution, which was then analyzed by the test strip with reflectometry method.

Flue-cured tobacco leaves: A 200 mg portion of samples (40–60 mesh) was accurately weighed into a 50 mL centrifuge tube. 40 mL of deionized water was added, and the mixture was manually shaken 70 times, then allowed to stand at room temperature for 36 min for static immersion extraction. After a second manual shaking of 70 times, a 2.0 mL aliquot of the extract was filtered through a hydrophilic membrane filter. A 200 µL portion of the filtrate was transferred to a 2 mL centrifuge tube, diluted to 2 mL with deionized water, and mixed thoroughly. The resulting solution was then analyzed by the test strip with reflectometry method.

### 2.4 Reflectometric analysis and glucose content calculation

The detection principle is based on measuring the reflected light intensity from a test strip, where glucose is oxidized by glucose oxidase to gluconic acid lactone, and the generated hydrogen peroxide reacts with an organic redox indicator in the presence of peroxidase to form a blue-green dye. Quantitative analysis was performed using a portable handheld reflectometer (RQFlex 20, Merck Company, Darmstadt, Germany). Prior to use, the instrument was turned on and allowed to warm up for 10 min. Calibration was carried out with a barcode strip and a blank test strip. The glucose measurement mode (range 1.00–100 mg/L) was selected, and the measurement was initiated by pressing the START key, which triggered a 60 s countdown on the reflectometer. Meanwhile, the two reaction zones of a glucose test strip (Merck, Cat. No. 1167200001) were immersed in the tobacco extract solution for 15–30 s, and excess liquid was allowed to drain off. During the last 10 s of the countdown, the test strip was inserted into the strip adapter with the reaction zones facing the instrument display. At the end of the countdown, the glucose concentration (mg/L) was read from the display. The RQflex 20 was cleaned and recalibrated after every 100 measurements. The glucose content (w, %) in fresh and flue-cured tobacco leaves was calculated using the equation: *w(%)* = C × D × V × 10^−4^/m × 100, where C is the glucose concentration in the extraction solution (mg/L), D is the dilution factor, V is the extraction solution volume (mL), and m is the weight of tobacco leaves (g). In the present study, the values of D, V, and m were 1 and 10, 100 mL and 40 mL, and 1.0 g and 0.2 g for fresh and flue-cured tobacco leaves, respectively.

### 2.5 Derivatization GC-MS method

A 50 mg portion of freeze-dried fresh tobacco or flue-cured tobacco samples was accurately weighed, and 30 μL of phenyl-*β*-D-glucopyranoside internal standard solution was added. After extraction, an aliquot of 100 μL of the extract was taken and dried prior to derivatization. The remaining extraction and derivatization procedures were performed according to the reference (Jing et al., 2024). This method can simultaneously detect diverse water-soluble sugars, including glucose, sucrose, maltose, etc.

GC-MS analysis was performed using an Agilent 7890A GC coupled with a 5975C inert XL mass selective detector (Agilent Technologies, Palo Alto, CA, United States), equipped with an HP-5ms capillary column (60 m × 0.25 mm i.d., 0.25 µm film thickness). The injection conditions were: split mode at 280 °C, injection volume 1 μL, split ratio 10:1. Helium (1.0 mL/min, constant flow) was the carrier gas. The column oven was programmed as: 60 °C (2 min), then 5 °C/min to 230 °C (5 min), then 8 °C/min to 290 °C (11.5 min). The transfer line temperature was set at 280 °C, and the solvent delay was 12 min. The ion source and quadrupole temperatures were 230 °C and 150 °C, respectively. Electron ionization (EI) was performed at 70 eV, and full-scan mass spectra were acquired over the range of m/z 45–600 for compound identification. The derivative products of glucose, phenyl-*β*-D-glucopyranoside, sucrose, and maltose were identified as MEOX-5TMS (36.998 min and 37.437 min), 4TMS (46.551 min), 8TMS (52.089 min), and MEOX-8TMS (53.803 min and 54.142 min), respectively. Quantification was performed in selected-ion monitoring (SIM) mode, with the quantifier ions set at *m/z*319, *m/z*361, *m/z*361, and *m/z*361, and the qualifier ions at *m/z*205 and 160; *m/z*271 and 243; *m/z*437 and 451; *m/z*191 and 281, respectively.

### 2.6 Data analysis

The extraction optimization using RSM-based central composite design (CCD) was carried out using Design-Expert software (Stat-Ease Inc., Minneapolis, MN, United States). Analysis of variance (ANOVA) was performed on the quadratic response surface model to validate its adequacy. Goodness-of-fit was assessed by the model *F*-value, *p*-value, lack-of-fit *p*-value, coefficient of variation (CV), and *R* ^*2*^. The criteria with *p* < 0.05, lack-of-fit > 0.05, CV < 10%, and *R* ^*2*^ > 0.90 were all satisfied, confirming that the model was reliable. A quadratic polynomial model was fitted using coded variables to predict the response at given levels of each factor. The relationship between the glucose content obtained via the two methods was assessed by Pearson correlation and simple linear regression, using SPSS 16.0 (SPSS Inc., Chicago, IL, United States). The AGREEprep evaluation was conducted with an open-source GUI-based software (available at git.pg.edu.pl/p174235/agreeprep). All other graphs were generated with Origin 2021 (OriginLab Corp., Northampton, MA, United States).

## 3 Results and discussion

### 3.1 Optimization of glucose extraction conditions

Various solvent systems, including water, acetonitrile, aqueous ethanol, and low-concentration sodium hydroxide solution, have been reported for the extraction of water-soluble sugars from tobacco (Jiang et al., 2019). In our study, the choice of extraction solvent must be compatible with the test strip-based detection system. The test strips contain a coated stationary phase that is susceptible to dissolution by organic solvents (e.g., acetonitrile and ethanol), which would cause severe interference; therefore, organic solvents were excluded. Additionally, the optimal pH range for the test strip assay is 2–10. Tobacco aqueous extracts are naturally slightly acidic (pH 4–6) due to the presence of organic acids such as citric and malic acids, which fall within this optimal range. Consequently, the use of low-concentration sodium hydroxide for extraction and pH adjustment is not required. To sum up, to meet the requirements of green chemistry principles, water was selected as the extraction solvent.

For fresh tobacco leaves, the grinding and homogenization process thoroughly disrupted the cellular structure, which significantly enhanced mass transfer efficiency and allowed rapid release of glucose into the aqueous phase. Hence, further extraction optimization was not required. Moreover, considering that the flue-cured tobacco matrices are more complex, the evaluation of disaccharide stability and method specificity was only conducted in the flue-cured tobacco matrices (Paul et al., 2025). For flue-cured tobacco, in order to meet the requirements of on-site rapid determination, manual shaking combined with static immersion extraction was adopted. Owing to the small sample size (0.2 g) and the relatively large volume of extraction solvent (40 mL), the liquid-to-solid ratio was sufficiently high, which had little effect on extraction efficiency. Thus, the effect of the liquid-to-solid ratio was not further investigated.

In preliminary experiments, single-factor tests were conducted to evaluate the effects of manual shaking count (10–90 times), static immersion time (5–45 min), and extraction temperature (4 °C–35 °C) on glucose extraction yield. The results indicated that shaking count and static immersion time had significant effects, whereas temperature exhibited only a minor influence. To obtain higher extraction efficiency while reducing the number of experimental runs, a multivariate optimization strategy, DoE-based RSM, was employed, using a two-factor, three-level CCD to optimize the shaking count (A, times) and static immersion time (B, min). CCD offers the advantages of evaluating interaction effects between factors, fitting quadratic responses, and requiring substantially fewer experiments than a full factorial design (Koronaiou et al., 2025). The CCD of coded variables, real variables, and the response factor of glucose extraction content is presented in Table 1. The coded levels were set as −1, 0, +1 and corresponded to the actual values of A (−1 = 22, 0 = 50, +1 = 78) and B (−1 = 11, 0 = 25, +1 = 39), respectively.

**TABLE 1:** CCD in coded variables, real variables, and response factors with glucose extraction content, and the main results of ANOVA for the fitted models.

Nos	Coded variables		Real variables		Response factors with glucose extraction content/%
	A	B	A/count	B/min	
1	0	0	50	25	8.87
2	0	−1.414	50	5	5.28
3	−1	−1	22	11	5.30
4	0	1.414	50	45	10.1
5	−1.414	0	10	25	6.82
6	1	−1	78	11	7.08
7	−1	1	22	39	8.93
8	0	0	50	25	9.01
9	0	0	50	25	8.88
10	0	0	50	25	8.86
11	0	0	50	25	9.05
12	1	1	78	39	10.2
13	1.414	0	90	25	9.38
ANOVA	*F*-value	*p*-value	​	​	​
A-A	529.3	<0.0001	Model *F* and *p*-value		612.5 and <0.0001
B-B	2189.5	<0.0001	Lack of fit *p*-value		0.29
AB	6.2	0.042	Coefficient of variation %		1.24
A^2^	117.6	<0.0001	Adjusted *R* ^*2*^		0.9961
B^2^	259.8	<0.0001	Adequate precision		72.65

The adequacy of the quadratic response surface model for glucose extraction content was verified by quadratic regression ANOVA in Table 1. The model proved to be highly significant, with an *F*-value of 612.5 and a *p*-value < 0.0001. All linear and quadratic terms, as well as the interaction between the two variables, were significant (*p* < 0.05). The adjusted coefficient of determination (*R* ^*2*^) was 0.9961, and the lack-of-fit *p*-value was 0.29 (>0.05), indicating that the lack of fit was not significant relative to pure error. A low CV (1.24%) indicated good reproducibility. The resulting quadratic polynomial model in terms of coded variables (A and B) is given by Equation 1:

$$R=8.93+0.8338A+1.70B-0.1275\text{AB}-0.4214A^{2}-0.6264B^{2}$$ (1)

**FIGURE 1:**
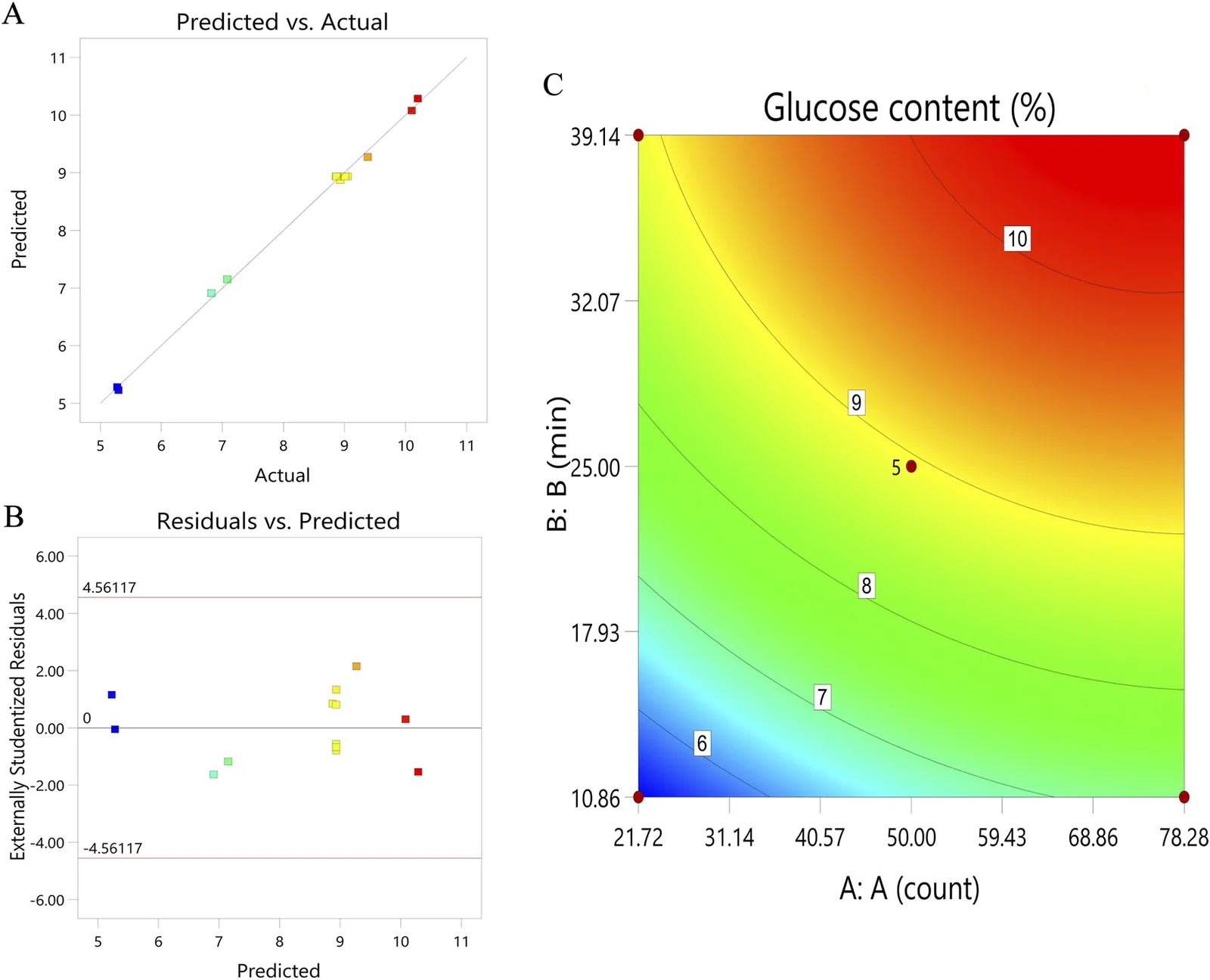
Results of a CCD for manual shaking count and static immersion time on glucose extraction content in tobacco: **(A)** Plot of the actual versus predicted values; **(B)** Plot of the externally studentized residuals versus the predicted values; **(C)** Contour plot of glucose extraction content.

Among them, R represents the glucose extraction content. As shown in Figure 1A, the predicted values from the model correlated well with the experimental values, with all data points in close proximity to the straight line, reflecting high accuracy. Figure 1B presents the plot of externally studentized residuals versus predicted values. The residuals were randomly scattered around zero, suggesting no violation of model assumptions (Kazmouz et al., 2025).

The final step was to determine the optimal value for each variable to achieve the maximum response. The contour map identified the optimal experimental parameters (Resende et al., 2025) in Figure 1C. Glucose extraction content increased significantly with both shake count and static immersion time. The maximum response (glucose content > 10%) was observed within the ranges of shake count 50–78 times and static immersion time 33–39 min. The optimal glucose extraction content was calculated with Equation 1 based on a total of 92 experimental runs. To minimize the overall extraction time, a higher shake count combined with a shorter static immersion time was selected. Under the conditions of 70 times and 36 min, the predicted glucose content was 10.2%. For model validation, three replicate experiments were carried out under the optimum conditions. No significant difference was found between the predicted and experimental values (10.3% ± 0.23%). Accordingly, the extraction conditions of 70 times and 36 min were adopted for all subsequent experiments. In consideration of potential deviations in shake count, the effect of 70–80 shakes on glucose extraction was further investigated. The results demonstrated that increasing the shake count exerted only a negligible influence on the extraction efficiency, indicating that extraction equilibrium had been reached at optimal conditions.

### 3.2 Evaluation of the disaccharide stability and optimization of the reaction time

**FIGURE 2:**
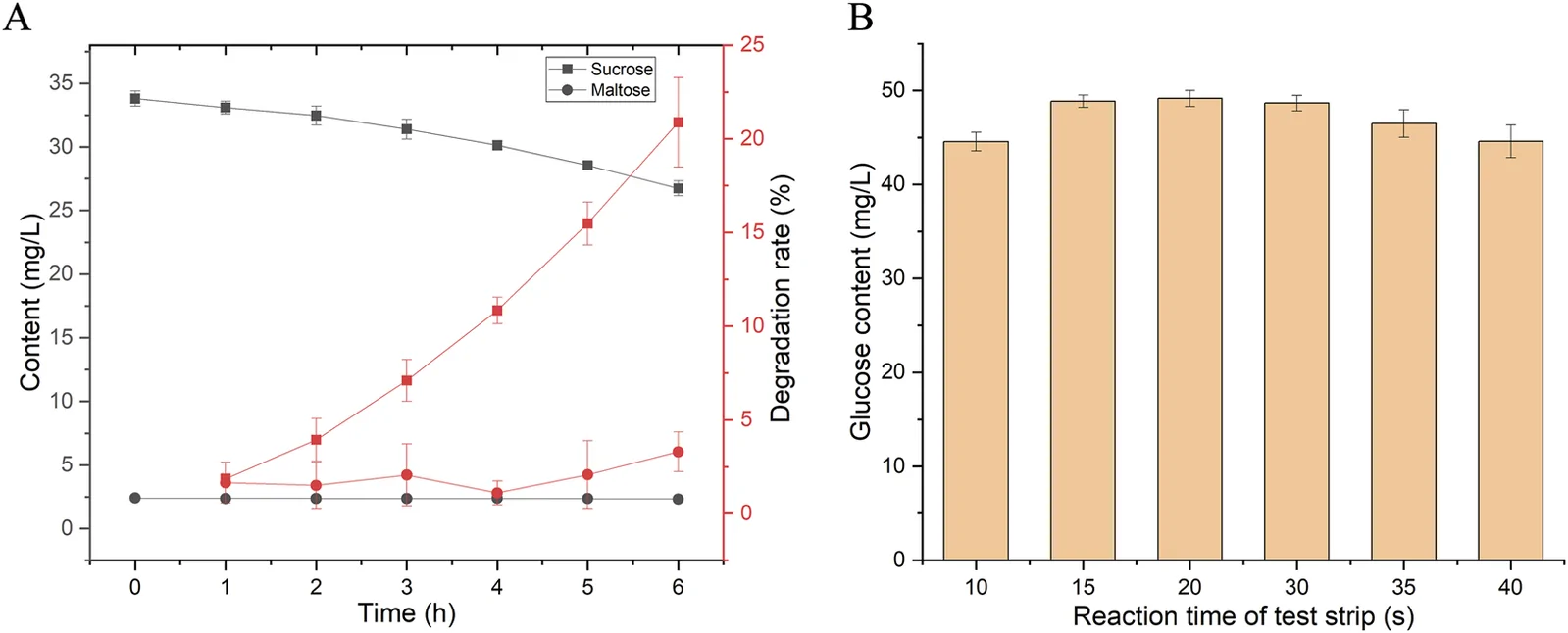
**(A)** Evaluation of changes in content and degradation rate of sucrose and maltose in aqueous extract of flue-cured tobacco with storage time; **(B)** Optimization of reaction time of test-strips in the aqueous extract.

The tobacco extract solution contained acidic substances such as citric acid, malic acid, and oxalic acid, which could catalyze the hydrolysis of sucrose and maltose into monosaccharides, thereby potentially interfering with the accurate quantification of glucose (Huang and Huang, 2012). pH measurement showed that a 0.25% (*w/v*) aqueous extract of flue-cured tobacco had a pH of 5.36. To evaluate the extent of sucrose and maltose hydrolysis in the aqueous extract, the residual amounts of sucrose and maltose were determined by derivatization-GC-MS at 0, 1, 2, 3, 4, 5, 6, 7, and 8 h of storage time. In Figure 2A, the results showed that the degradation rate of sucrose was less than 4% within 2 h, but increased significantly with prolonged storage time, which was likely attributable to the gradual decrease in pH of the extract over time (Li et al., 2003). In contrast, maltose remained essentially undegraded over time. Literature reports indicate that the acid-catalyzed hydrolysis reactivity of sucrose is much higher than that of maltose, which is attributed to the greater lability of the α-1, 2-glycosidic bond in sucrose compared to the α-1, 4-glycosidic bond in maltose (Marzo et al., 2011; Vilcocq et al., 2014). Since the test strip with reflectometry method allows rapid determination (1–2 min per sample), the potential hydrolysis of sucrose does not interfere with glucose quantification under the present conditions.

For reflectometric determination, the reaction time (10, 15, 20, 30, 35, and 40 s) of the test strip in the sample solution was optimized. As shown in Figure 2B, accurate and stable results were obtained within the reaction time range of 15–30 s. An insufficient reaction time (<15 s) resulted in incomplete wetting of the reaction zones and consequently low glucose content, whereas an excessive reaction time (>30 s) caused partial dissolution of the coated stationary phase, also leading to low content (Major and Barraclough, 2003). Therefore, the reaction time of 15–30 s was adopted.

### 3.3 Method matrix effect and specificity

A study was conducted to evaluate the interference degree in the determination of glucose in fresh and flue-cured tobacco matrices using matrix effect and specificity. The matrix interference factor (*f*) was introduced as an evaluation criterion (Cai et al., 2010). The *f* was defined as the ratio of the glucose concentration measured in the matrix to the real spiked concentration. By spiking the tobacco matrix with standard solutions at low and high glucose concentration levels, the *f* in the tobacco matrix was obtained as follows: *f* = (C_spiked_ - C) / RC, where C_spiked_ is the concentration determined after spiking, C is the concentration determined without spiking, and RC is the real spiked concentration. According to the *f* definition, an *f*-value greater than 1 indicates signal enhancement, an *f*-value equal to 1 indicates no matrix interference, and an *f*-value less than 1 indicates signal suppression. The results are presented in Table 2. The *f*-values for glucose in tobacco samples using test strip with reflectometry method were all less than 1, indicating a matrix-induced signal suppression effect. However, the relative errors caused by the matrix effect were all between 2.0%–5.0%, suggesting that the suppression effect was not significant and the tobacco matrices did not appreciably affect the glucose quantification (Cai et al., 2012).

**TABLE 2:** Matrix interference factor of glucose in different tobacco matrices.

Matrices	RC/mg/L	C_spiked_/mg/L	C/mg/L	*f*	Relative error
Flue-cured tobacco	20.1	69.4	50.1	0.961	3.9%
	40.1	87.7	49.6	0.950	5.0%
Fresh tobacco	15.0	53.2	38.5	0.980	2.0%
	30.0	67.6	39.0	0.953	4.7%

Similar to electrochemical sensing techniques, the test strip with reflectometry method relies on a specific response to glucose for accurate quantification. However, this specificity can be compromised by co-existing metabolites that are structurally analogous to glucose, present at high concentrations, or capable of altering the sample solution pH (Özbek and Şahin, 2025). Notably, although matrix effect evaluation can reflect the overall influence of the entire sample matrix on glucose quantification for a given batch of samples, the contents of various chemical constituents in tobacco (e.g., organic acids, sugars, alkaloids) vary considerably depending on factors such as cultivar, region, and growth stage. Consequently, matrix effect evaluation based on a limited number of samples may not fully cover all possible matrix compositions. Therefore, specificity evaluation is necessary to investigate those representative metabolites that cause potential interference. To this end, fructose, sucrose, myo-inositol, malic acid, and nicotine were selected as representative metabolites with structural similarity or high abundance. Their effects on glucose determination were assessed by comparing the results obtained from blank samples and samples spiked with each metabolite at three concentration levels (low, medium, and high) in the flue-cured tobacco extract. The results are presented in Table 3. The mean glucose concentrations ranged from 49.4 to 51.5 mg/mL, with relative errors between −2.0% and 2.2%, indicating that none of these metabolites significantly affected the glucose quantification. All these findings collectively demonstrate that the proposed test strip with reflectometry method exhibits low matrix effects and high selectivity, which makes it suitable for accurate on-site determination of glucose content in tobacco matrices.

**TABLE 3:** The influence of tobacco co-existing metabolites on method specificity.

Spiked concentration/mg/L	Fructose	Sucrose	Myo-Inositol	Malic acid	Nicotine
0	50.4				
20	48.9	51.3	49.3	50.8	52.6
40	49.9	49.6	50.4	48.2	51.2
60	49.5	50.6	52.1	49.3	50.6
Average	49.4	50.5	50.6	49.4	51.5

### 3.4 Method validation

The analytical performance of the test strip with reflectometry method was evaluated with linearity, sensitivity, accuracy, and precision. A solvent-matched calibration curve was constructed over the concentration range of 2.00–90.0 mg/L using a weighted (1/X) least-squares linear regression. The regression equation was y = 0.9952x + 0.0253, with a correlation coefficient (*R*) of 0.9991. Based on the sample weight, dilution factor, and recovery, the sample measured ranges were calculated as 0.21%–20.58% for flue-cured tobacco and 0.011%–1.03% for fresh tobacco leaves, which adequately covered the typical glucose levels found in tobacco leaves. If the sample falls outside this range, the extract is to be appropriately concentrated or diluted.

The accuracy of the method was evaluated through recovery experiments. The recovery percentage (R, %) was calculated using the formula: R (%) = (C _detected_-C _sample_) × 100/C _spiked_, where C _detected_ and C _sample_ are the glucose concentrations in the spiked and unspiked samples, respectively, and C _spiked_ is the known concentration added. Recovery was determined at both low and high spiking levels, with five replicate measurements per level. Precision was assessed in terms of repeatability (intra-day) and reproducibility (inter-day). Repeatability was expressed as the relative standard deviation (RSD) of five consecutive measurements performed on the same day, while reproducibility was evaluated as the RSD of measurements conducted on three consecutive days. The results are summarized in Table 4. The average recoveries of glucose ranged from 94.1% to 96.0% for flue-cured tobacco leaves with RSDs for repeatability (1.85%–3.86%) and reproducibility (2.86%–5.00%), and from 95.0% to 97.5% for fresh tobacco leaves with RSDs for repeatability (2.39%–4.51%) and reproducibility (3.84%–5.98%). These data demonstrate that the developed method meets the validation criteria recommended by the International Council for Harmonisation (ICH) guidelines (Mestareehi, 2025), providing satisfactory linearity, accuracy, and precision for rapid on-site glucose quantification in tobacco matrices.

**TABLE 4:** Recovery, repeatability, and reproducibility of glucose analysis in flue-cured tobacco and fresh tobacco leaves using the test strip with reflectometry method.

Matrices	Content/%	Spiked content/%	Detected content/%	Recovery/%	Repeatability/%	Reproducibility/%
Flue-cured tobacco	9.95	4.01	13.8	96.0	3.86	5.00
		8.02	17.5	94.1	1.85	2.86
Fresh tobacco	0.38	0.20	0.57	95.0	4.51	5.98
		0.40	0.77	97.5	2.39	3.84

### 3.5 Comparison of accuracy and greenness between the silylation derivatization GC-MS and the test-strip with reflectometry method

**FIGURE 3:**
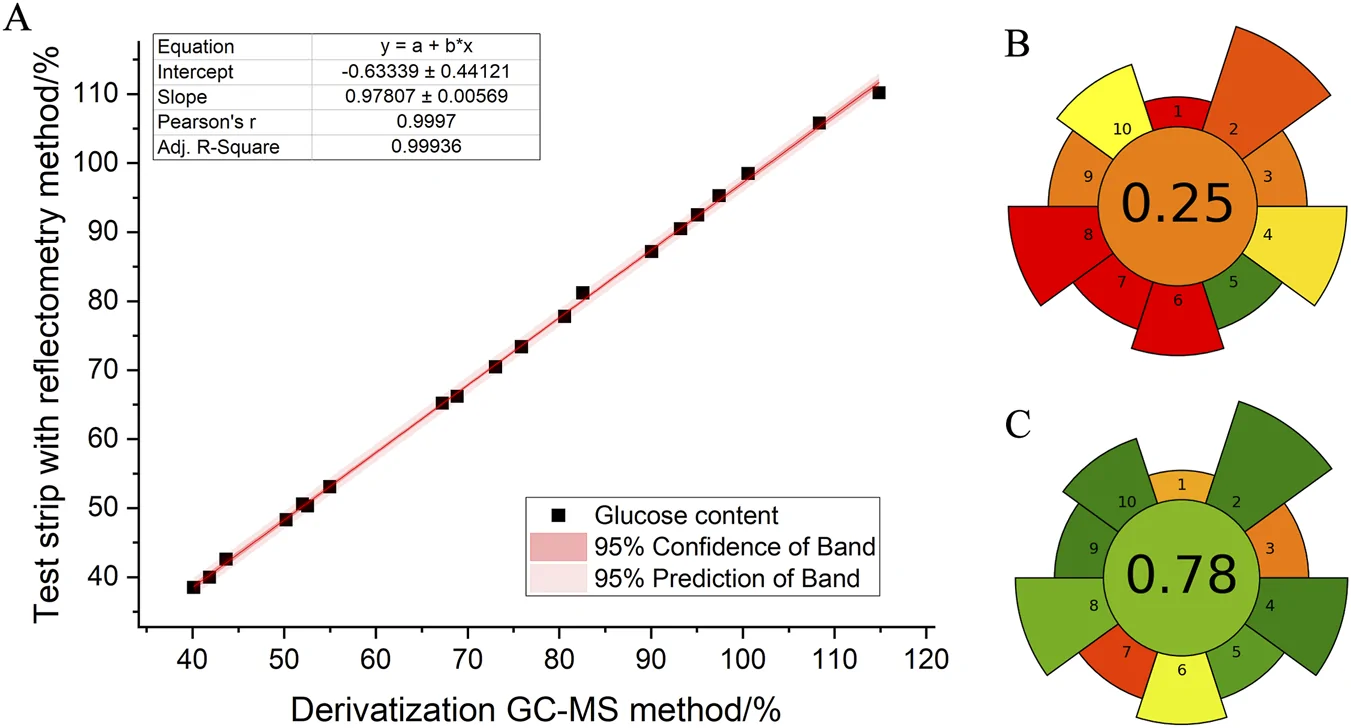
**(A)** Relationship analysis between the test strip with reflectometry and derivatization GC-MS method; **(B)** The overall AGREEprep score of the derivatization GC-MS method; **(C)** The overall AGREEprep score of the test strip with reflectometry method.

To further validate the accuracy, twenty tobacco samples were determined by both the test-strip with reflectometry and the silylation derivatization GC-MS method, and the results were compared with linear relationship analysis. As shown in Figure 3A, the correlation between the two methods was highly significant (*p* < 0.01), with Pearson *r* = 0.9997. The linear regression equation was y = 0.9781x - 0.6334 with *R* ^*2*^ = 0.9994, indicating that the two methods agreed well with a 1:1 relationship and further confirming the comparable accuracy. The slope is slightly less than 1, which can be attributed to the matrix suppression effect in the test-strip method, whereas the silylation derivatization method reportedly suffers from a matrix enhancement effect (Cai et al., 2012). The resulting slope error was 2.2%, and the overall error was less than 5%. Given the small discrepancy, this difference does not affect the results of the tobacco leaf quality evaluation. Previous studies have also compared reflectometers with HPLC for the determination of nitrate and 5-(hydroxymethyl)furan-2-carbaldehyde (HMF) in soil and beer (Vrzal et al., 2019; Yue et al., 2012). The reflectometer showed excellent agreement with the laboratory HPLC method for covering a wide range of climatic and geographic regions, soil types, croplands, and soil texture matrices in nitrate. The linear regression had slopes of 1 ± 0.08 and intercepts of ±1.38. For HMF, the linear relationship for both methods was significant, but the slope was slightly lower at 0.912, which may be attributed to interference from furan-2-carbaldehyde. Nevertheless, the quantitative results are sufficiently reliable for evaluating the stale flavor quality of beer. Recently, the portable RQflex has been used for field analysis of nitrate concentrations in springs and streams in the Pukekohe region. The results clearly reflected contamination levels and water quality, and flexible field measurements enabled to collect water samples at proper distances without missing crucial information (Rogers et al., 2025). Overall, the test-strip with reflectometry method exhibited accuracy comparable to that of the silylation derivatization GC-MS method in glucose analysis, and previous studies also demonstrated that this method exhibits a significant correlation with conventional analytical methods and shows favorable compatibility in field application. In addition, the portable reflectometer offers advantages such as ease of use, low cost, and good portability, making it suitable for on-site rapid detection.

To objectively evaluate the environmental impact of our analytical protocol, the recently reported AGREEprep metric was employed, as it specifically emphasizes the compatibility between sample preparation and instrumentation. The method is assessed against ten criteria (Wojnowski et al., 2022): 1) Sample preparation and placement; 2) Toxic materials; 3) Sustainable and renewable materials; 4) Waste; 5) Size economy of the sample; 6) Sample throughput; 7) Integration and automation; 8) Energy consumption; 9) Post-sample preparation configuration for analysis; 10) Operator safety. Each criterion is scored from 0 (worst) to 1 (best), and the weighted sum of these scores gives the overall performance. The final assessment is presented as a colorful circular pictogram featuring a central numeric score, and the inner-circle color, along with the score, reflects the overall greenness of the sample preparation procedure (Pena-Pereira et al., 2022).

The test strip with reflectometry method exhibits a superior AGREEprep score (0.78) compared to the derivatization GC-MS method (0.25) in Figures 3B,C. The improved environmental performance of the test strip with reflectometry method is primarily attributed to excellent performance in criteria 2, 4, 9, and 10. More specifically, this method can operate with water extraction and does not involve any harmful solvents. The amounts of hazardous materials and waste are basically negligible, so there is virtually no risk of chemical exposure and acute toxicity with good operational safety. The type of instrumentation is test strips and handheld reflectometry, which are characterized by their simplicity, rapidity, and on-site analysis. In addition, criteria 5 and 8 also show good environmental performance. Overall, the AGREEprep assessment demonstrates test strip with reflectometry method significantly improves its environmental sustainability, in accordance with the principles of green chemistry (de Souza et al., 2026). However, it should be noted that the derivatization GC-MS method enables comprehensive profiling analysis of dozens of sugars (e.g., fructose, sucrose, inositol, etc.); its overall greenness warrants further optimization and assessment.

### 3.6 Sample analysis of different tobacco cultivars and growth stages

**FIGURE 4:**
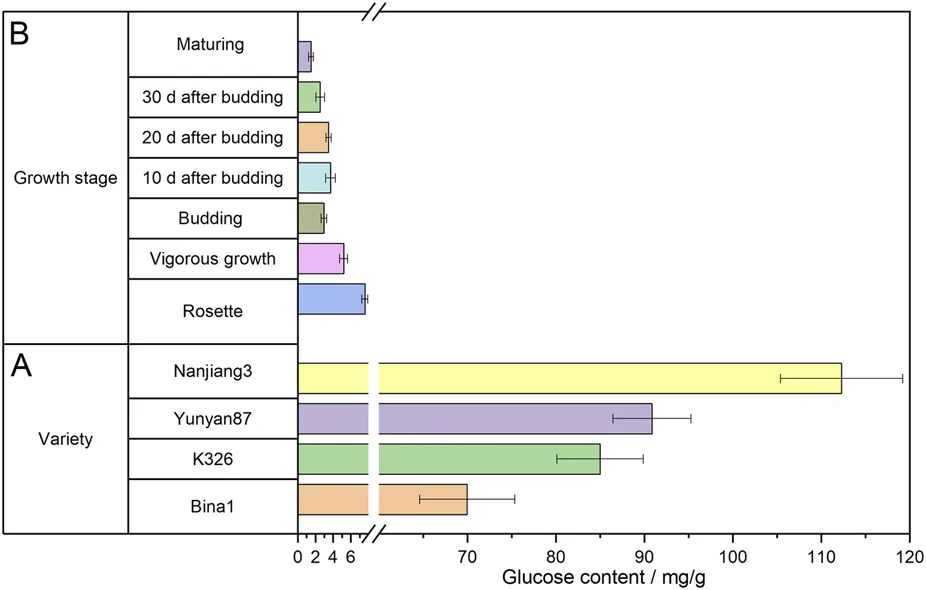
**(A)** Comparison of glucose contents in different flue-cured tobacco cultivars (Nanjiang3, Yunyan87, K326, and Bina1); **(B)** Variation of glucose content in fresh leaves of Yunyan87 at seven key growth stages.

The test strip with reflectometry method was applied to the flue-cured tobacco of four cultivars (Nanjiang 3, Yunyan87, K326, and Bina 1) and fresh tobacco leaves of seven growth stages (Yunyan87) to monitor the glucose content on-site. As shown in Figure 4A, the glucose content was highest in Nanjiang 3, followed by Yunyan 87 and K326, while Bina 1 exhibited the lowest content. This variation reflects the genetic differentiation in carbon metabolic allocation among different flue-cured tobacco cultivars during growth and curing. In this study, it was found that the glucose content in fresh leaves of Yunyan 87 (on a dry weight basis) accounted for only 8.43% of that in the corresponding cured leaves, indicating that the hydrolytic conversion of starch during the curing process is a critical determinant of glucose accumulation in tobacco leaves (Zong et al., 2022).

The changes in glucose content in fresh leaves of Yunyan 87 at seven key growth stages are also shown in Figure 4B, exhibiting a trend of the highest content at the rosette stage, followed by a decline during the vigorous growth/budding stage, a transient increase after budding, and a continuous decrease to the lowest level at maturity. The rosette stage, characterized by vigorous vegetative growth, exhibited active carbohydrate synthesis and then glucose accumulation at peak. Subsequently, glucose content gradually decreases due to the shift of the growth center and the increase in metabolic consumption at the vigorous growth and budding stages. At 10 days after budding, the release of apical dominance and redistribution of photosynthates lead to a transient increase in glucose content. Thereafter, as leaf senescence and dry matter conversion proceed, the glucose content continues to decline, reaching 1.52 mg/g (Drapal et al., 2021; Liu et al., 2022). These results indicated that the developed method was efficient and well suited for on-site glucose determination in both flue-cured and fresh tobacco leaves.

## 4 Conclusion

In this study, a simple, portable, and green on-site method was established for the quantitative determination of glucose in both flue-cured and fresh tobacco leaves, based on aqueous extraction followed by test strip reflectometric analysis. RSM-based CCD was employed to optimize the shaking count and static immersion time, achieving better extraction efficiency, thereby meeting the operational requirements of rapid on-site detection. To assess potential matrix interferences, it was confirmed that hydrolysis of sucrose and maltose in the tobacco aqueous extract did not significantly affect the glucose content within 2 h, and sucrose was much more susceptible to hydrolysis than maltose. The matrix effect was found to be suppressive, but its impact on the quantitative results was negligible. Selectivity evaluation demonstrated high specificity in the enzymatic reaction, with no significant interference from major co-existing metabolites in tobacco. Method validation and comparison confirmed that the developed method offers high accuracy and good repeatability, with a measuring range covering the glucose content variation in the vast majority of tobacco samples. The greenness assessment indicated that this method significantly enhances its environmental sustainability, consistent with green chemistry principles. The method application revealed that glucose content in fresh tobacco leaves (on a dry weight basis) accounted for less than 10% of that in the corresponding cured leaves and exhibited fluctuation across different growth stages, with the highest level observed at the rosette stage. In summary, the proposed method enables simple, accurate, and green on-site determination of glucose in tobacco leaves, demonstrating considerable practical applicability and potential for routine use. Despite this method offering certain advantages, it suffers from the limitation of a relatively narrow measuring range and can only detect one sugar species for quality evaluation, whereas comprehensive tobacco quality may be more influenced by the synergistic effects of various water-soluble sugars.

## Data Availability

The original contributions presented in the study are included in the article/supplementary material, further inquiries can be directed to the corresponding authors.
